# Stone extraction during flexible ureterorenoscopy with or without the hydrogel method: a randomized, multicenter safety and feasibility study

**DOI:** 10.1007/s00345-025-06011-7

**Published:** 2025-10-31

**Authors:** T. Amiel, A. Neisius, C. Netsch, A. Secker, C. Fisang, I. Grunwald, L. Lunger, M. Straub

**Affiliations:** 1 Department of Urology, Technical University of Munich, School of Medicine and Health, Munich, Germany; 2 Department of Urology, Clinic Barmherzigen Brüder Trier, Trier, Germany; 3 Department of Urology, Asklepios Klinik Barmbek, Hamburg, Germany; 4 Department of Urology, University Hospital Münster, Münster, Germany; 5 Department of Urology, Marienhaus Klinikum, Bad Neuenahr-Ahrweiler, Germany; 6 Department of Industrial and Environmental Biology, Hochschule Bremen, City University of Applied Sciences, Bremen, Germany

**Keywords:** Urolithiasis, Flexible ureterenoscopy, Hydrogel method, Stone dusting, Stone free rate

## Abstract

**Purpose:**

Achieving the highest possible stone-free rate is the primary goal of kidney stone surgery, yet standard flexible ureterorenoscopy often leaves small residual fragments that predispose to recurrence. The hydrogel method using mediNiK^®^ was developed to embed and extract even the smallest fragments with conventional baskets. This study aimed to evaluate the safety and feasibility—defined as the ability to extract fragments < 1 mm—of stone extraction during flexible ureterorenoscopy with or without the hydrogel method.

**Materials and methods:**

This prospective, randomized, multicenter proof-of-concept trial included patients > 18 years with kidney stones > 8 mm and no anatomical abnormalities. Patients were randomized to flexible ureterorenoscopy + hydrogel (Group 1) or flexible ureterorenoscopy (Group 2) alone. After laser lithotripsy, fragments were retrieved either embedded in hydrogel or individually, and categorized by size (< 0.5 mm, 0.5–1.0 mm, > 1.0 mm). Explorative statistical analyses included Mann-Whitney U, Student’s t-test, and Chi-square test. Adverse events were monitored intraoperatively and during a 6-week follow-up.

**Results:**

Of 65 screened patients, 40 were analysed (Group 1: *n* = 23; Group 2: *n* = 17). The hydrogel method significantly retrieved more fragments < 1 mm (1716 vs. 209) and > 1 mm (310 vs. 118). On a per-patient level, more < 1 mm fragments were removed in Group 1 (median 7 [IQR 21] vs. 0 [2], *p* < 0.003). Surgery duration was longer in Group 1 (80 [28] vs. 62 [20] minutes, *p* = 0.02). No serious adverse events were reported.

**Conclusions:**

The hydrogel method was a safe and feasible addition to fURS, allowing improved retrieval of even the smallest fragments without added risk. The main limitations are the small sample size and absence of long-term stone-free and recurrence data, underscoring the need for larger confirmatory studies.

## Introduction

The global incidence of kidney stones has risen, especially in Western industrialized nations [1,1]. Parallel to this, treatment has evolved from open surgery to extracorporeal shock wave lithotripsy (SWL) [2], to minimally invasive techniques like percutaneous nephrolithotomy (PCNL) and flexible ureteroscopy (fURS) [2, 3]. These advances have been supported by improvements in endoscopes, materials, and lasers [4, 5]. Despite these innovations, residual fragments post-treatment remains challenging. Once considered harmless, clinically irrelevant residual fragments (CIRF), typically 2–4 mm in size, can persist without spontaneous passage, leading to recurrence or inaccessibility with standard instruments [6–9]. To address this, maximizing the stone-free rate (SFR) is essential [10]. Traditional methods like the autologous blood clot technique (ABC), also known as coagulum pyelolithotomy, have been used [11]. Newer methods in combination with different type of suction such as have been developed and are becoming more popular aswell. A novel approach, the hydrogel method (HM) using mediNiK^®^ (MN), shows promise. This involves injecting a biocompatible hydrogel into the calyx to encapsulate fragments. Unlike ABC, HM provides controlled entrapment, conforms to calyceal contours, is transparent and gradually degrades, minimizing complications and facilitating fragment elimination. Preclinical testing—both in vitro [12, 13] and in porcine vivo [14]—has confirmed feasibility. The first human use was reported in September 2021 [15]. This study aimed to assess the safety and feasibility of stone extraction using HM with MN versus standard of care (SoC) in a broader patient cohort.

## Patients and methods

### Study design, setting and study population

This single blinded prospective study was carried out between September 2021 and September 2022 on patients scheduled for an elective fURS with lithotripsy at five German hospitals (Technical University Hospital of Munich, Clinic Barmherzige Brüder of Trier, Asklepios Klinik Barmbek in Hamburg, University Hospital of Münster, Clinic Maria Hilf in Bad Neuenahr-Ahrweiler). All patients with confirmed kidney stones larger than 8 mm on preoperative computed tomography (CT) were considered eligible for participation. A cutoff of 8 mm was chosen, as larger stones are unlikely to pass spontaneously, are difficult to extract intact during ureterorenoscopy, and typically require lithotripsy.

Exclusion criteria encompassed individuals who were unable to provide informed consent, untreated urinary infections, anatomic abnormalities (e.g. solitary renal, untreated ureteral stricture, ureteropelvic junction obstruction), tumors in the urinary tract, cases where stone extraction was performed without laser lithotripsy, and instances where the kidney could not be reached with the ureteroscope.

### Description of the medical product

**Fig. 1:**
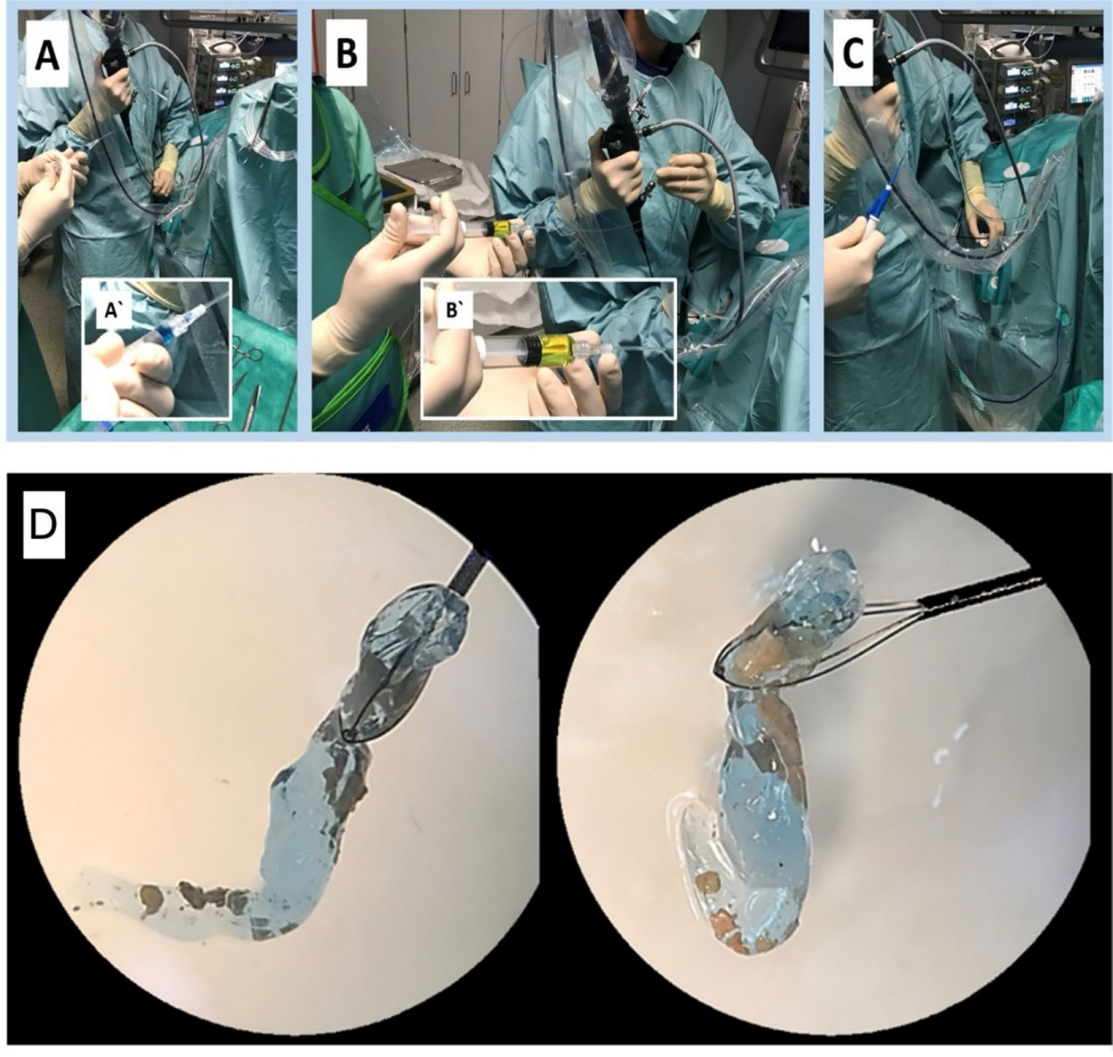
Application of mediNiK^®^, **A** application of K1, **B** application of K2, **C** extraction of the hydrogel clot, **D** extracted hydrogel clot embedding stone fragments

The medical product used for the HM is mediNiK^®^, a Class I (sterile) medical device according to the European Union medical device classification (Directive 93/42/EEC1). CE certification was granted in May of 2021. MN consists of two biocompatible liquid components (K1 – Alginate and K2 – Calcium ions) pre-filled in syringes. K1 is applied over the stone fragments following laser lithotripsy through the flexible endoscope working channel. The addition of K2 to K1 results in the spontaneous and rapid formation of a hydrogel embedding the fragments. Finally, the gel clot can be removed with a basket (Fig. 1). If needed, the hydrogel complex can be dissolved with saline. Retrieving all gel fragments at the end of the procedure is not mandatory, as they will dissolve with the patient’s urine within 60 min. All participating surgeons were inexperienced with MediNiK^®^-assisted fURS before the start of this study.

### Surgery and randomization

fURS were performed as follows: a safety guide wire was placed into the renal pelvis after the initial cystoscopy and a standard ureteral access sheath (UAS) (size 10/12 or 12/14 Fr, length 35–45 cm) was employed to facilitate stone extraction. fURS was performed with either single-use or reusable flexible scopes, laser ( Ho: YAG or Tm: YAG) and laser fibers, according to center-specific standards. Upon reaching stones deemed too large for removal, a laser lithotripsy was executed in all patients. In the fURS alone group, the surgeon performed fragmentation (settings: 1,8–2,5 Joules, 3–8 Hz) combined with basket removal or popdusting (settings: 1,5–2,0 Joules, 12–18 Hz). For patients in the fURS + MN group, the surgeon performed dusting (settings: 0,3–0,7 Joules, 20 Hz) until stone fragments were sufficiently small, then applied MN (one application per patient). After the formation of the hydrogel embedding the fragments, it was extracted using a conventional basket.

Prior to the operation, randomization was electronically conducted to assign patients to either fURS in combination with MN or fURS alone. The surgeon was informed before the operation.

### Stone classification

Stone–gel mixtures were filtered with a 15 mL vacuum unit using 25 mm glass-supported cellulose nitrate membranes, rinsed three times with 5 mL water, and the hydrogel was dissolved in 5 mL of 0.2 M Na-EDTA by ten pipetting cycles. After vacuum removal and three additional rinses, filters with stones were dried at 40 °C for 3 h. Stones were size-fractionated (large/medium/small), imaged under a Stemi DV4 stereomicroscope (8×) with a calibration slide, and analyzed in ImageJ. The longest diameter of each fragment was measured and exported to calculate counts and mean size per fraction.

### Objectives, outcome measures and statistical overview

The primary objective of the study was to assess the feasibility and safety of stone extraction with fURS + MN compared to standard fUR. Extracted stones were classified by size (< 0.5 mm, 0.5 to 1 mm, > 1 mm) and treatment arm.

As a surrogate for feasibility of the MN method, each intervention’s potential to extract stone fragments smaller and larger than 1 mm (number of fragments) was evaluated. The 1 mm cut-off was chosen because smaller fragments are typically difficult to retrieve using a conventional basket and are often left behind as CIRF. Additionally, the duration of surgery (in minutes) and the subjective difficulty of stone removal, as rated by the surgeon on a 5-point Likert scale (0 = not difficult, 5 = very difficult), were recorded.

Safety was evaluated by monitoring adverse events during surgery throughout a post-operative period of six weeks. To illustrate the safety profiles of both groups, descriptive summaries of treatment-related adverse events (TEAEs) were provided, including absolute numbers, percentages, intensity, and relationship to MN, without statistical comparison.

For exploratory purposes, statistical analyses between groups were performed using Student’s *t*-test, Mann–Whitney U test, and Chi-square test to assess the preliminary efficacy of gel-assisted stone removal versus fURS alone on a per-patient basis, as well as surgical time and subjective difficulty of stone removal.

This prospective multicenter randomized study (DRKS00030532) was approved by local ethics committees in accordance with the Helsinki Declaration.

## Results

### Patient characteristics

**Fig. 2:**
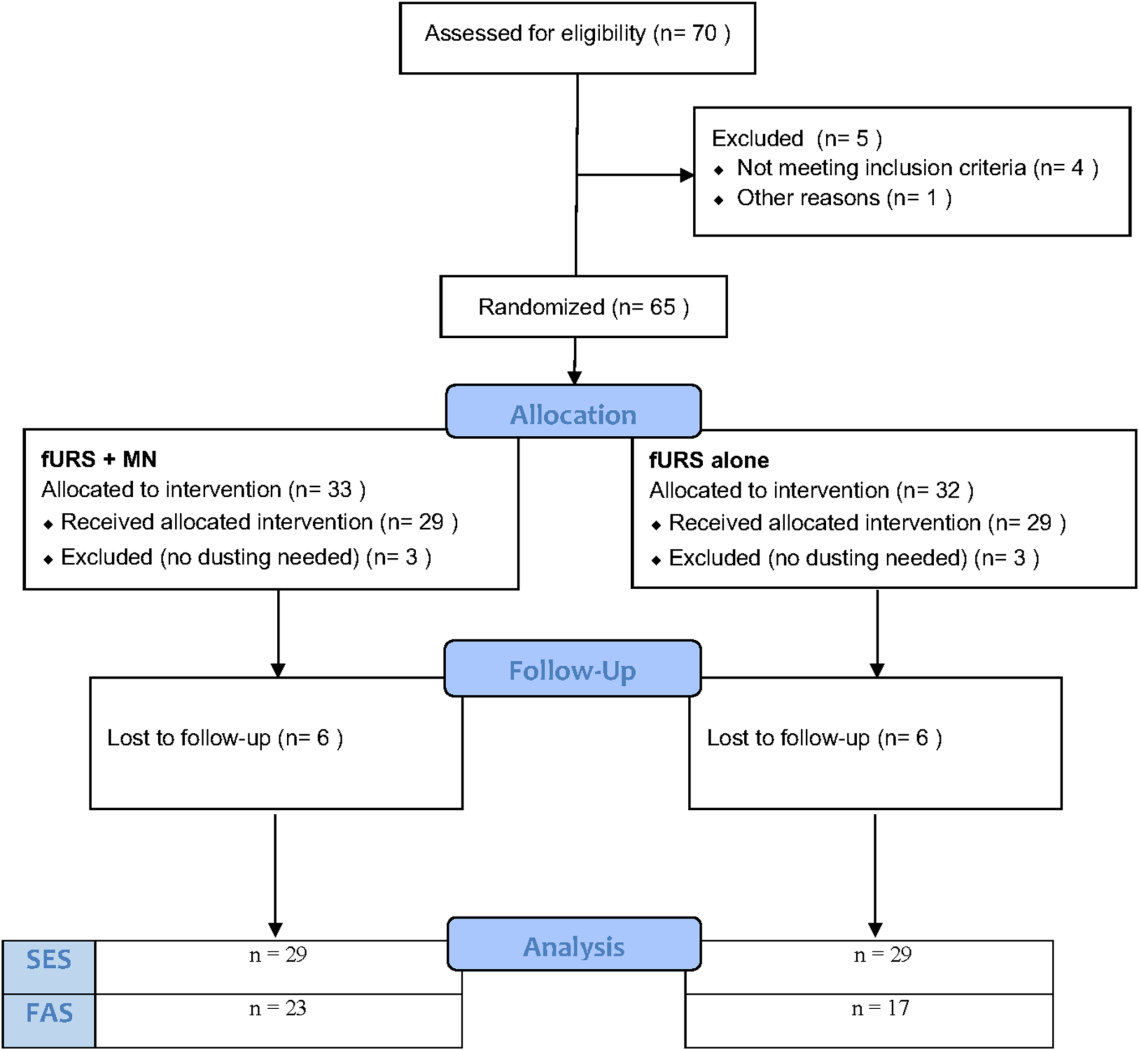
CONSORT FlowChart. *SES* safety evaluation set, *FAS* full analysis set

Seventy patients undergoing elective fURS lithotripsy were screened for this study between September 2021 and September 2022 at five hospitals in Germany. The baseline characteristics of patients in both groups are presented in Table 1. Figure 2 shows the CONSORT (Consolidated Standards of Reporting Trials) flow chart. Following randomization, UAS placement was successful in all cases, with no failures observed.

For this study, two analyses sets were considered: (1) Safety Evaluation Set (SES), which included all subjects with performed kidney stone removal procedure in either arm (with or without postoperative follow-up) and (2) Full Analysis Set (FAS) (all randomized subjects of the SES including a complete follow-up).

### Safety

The overall summary of treatment-emergent adverse events (TEAEs) for SES is shown in Table 2. Irrespective of the type of treatment received, there were no severe TEAEs in either treatment arm, and TEAEs of any grade were rare in this small cohort of patients. Overall, although not statistically tested, TEAEs appeared to occur more often in patients undergoing fURS alone (*n* = 7/29, 24.14%) versus those in the fURS + MN group (*n* = 5/29, 17.24%). Patients in the fURS group had numerically more TEAEs related to the urinary tract than those in the fURS + MN group. All TEAEs were Clavien-Dindo grade I–II.

### Feasibility

**Fig. 3:**
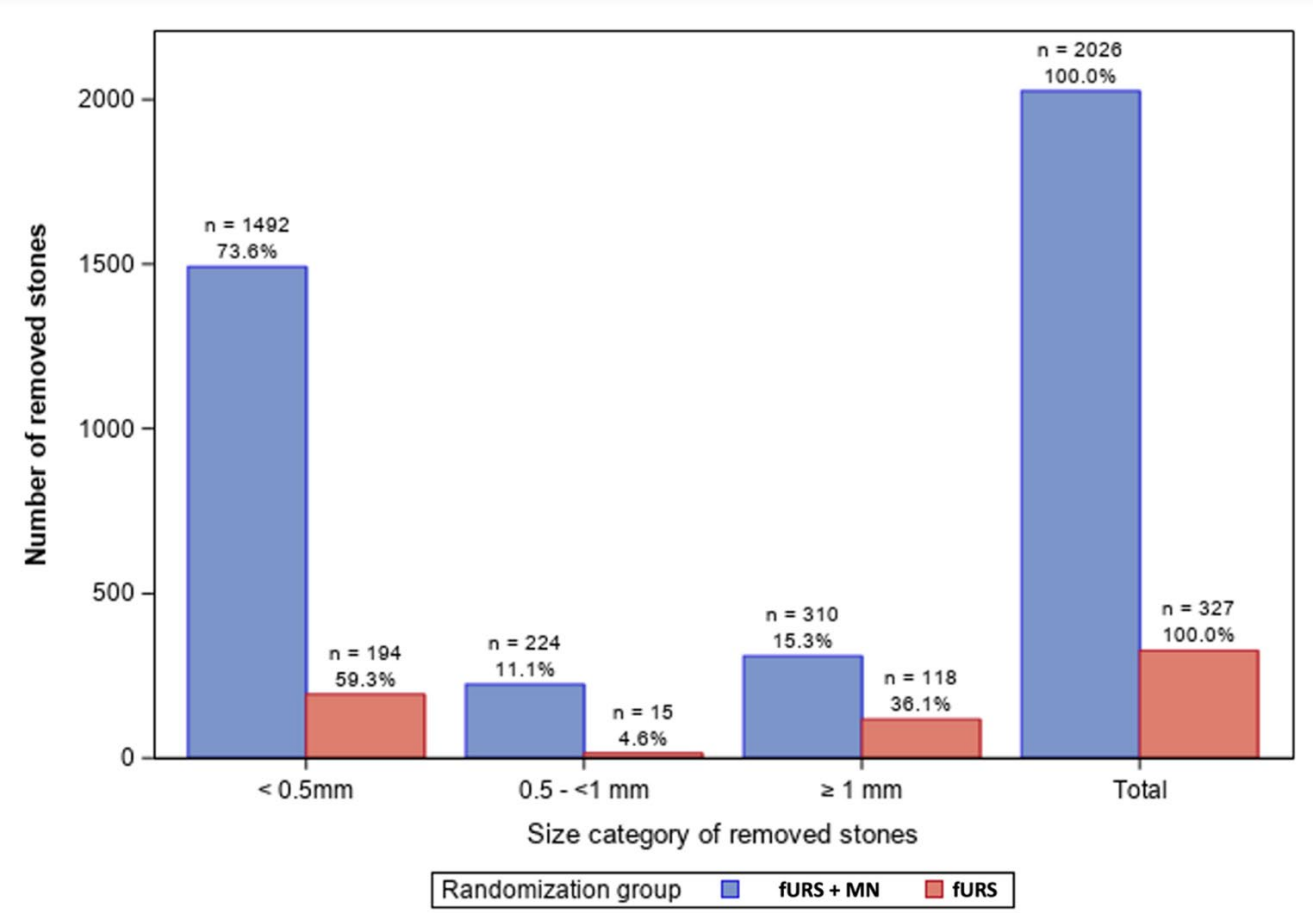
Bar chart of size of extracted kidney stones per treatment arm, *n* number of removed stones/fragments. *%* percentage based on the total number of extracted stones per treatment arm. *fURS* flexible ureteroscopy, *MN* mediNiK^®^

Figure 3 highlights the total number of fragments extracted per size category based on the Full Analysis Set (FAS), plotted by treatment type. Overall, considering smallest fragments < 1 mm, fURS + MN resulted in an approximately 8-fold higher number of stones < 1 mm extracted as compared to fURS alone (1716 versus 209 fragments, respectively). Similarly, for fragments > 1 mm, fURS + MN resulted in twice as many fragments extracted (310 vs. 118). On a per patient level (median (IQR)), a significantly higher median number (IQR) of 7 fragments (21) was extracted using fURS + MN as compared to fURS alone (0 fragments (2)), *p* = 0.013.

With regards to the duration of surgery, this study revealed a statistically longer median procedural time (IQR) for fURS + MN compared to fURS alone (80 min (28) versus 62 min (20), *p* = 0.02. Of note, despite the longer duration of fURS + MN, there was no difference with regards to the perceived difficulty of both interventions as determined by the performing surgeons between both groups (*p* = 0.29, Table 3).

**Table 1:** Baseline charateristics of patients in the safety ealuation set prior to surgery

Parameter	fURS + MN (*n* = 29)	fURS Alone (*n* = 29)
Age, yr (mean ± SD)	52,3 (± 15,9)	51.7 (± 16,5)
BMI (kg/m^2^) (mean ± SD)	27.6 (± 4.44)	28.6 (± 6.74)
Female, n (%)	10 (34.5%)	10 (34.5%)
Male, n (%)	19 (65.5%)	19 (65.5%)

*fURS* flexible ureteroscopy, *MN* mediNiK^®^, *BMI* body mass index

**Table 2:** Treatment-emergent adverse events across both treatment arms within the SES (*n* = 29 Pts per treatment arm)

	Treatment type	
	fURS + MN, % of total (*n*) (SES, *n* = 29)	fURS, % of total (*n*) (SES, *n* = 29)
No. Pts. with at least one TEAE (any grade)	17.2 (5)	24.1 (7)
Type and frequency of adverse events		
*Related to the urinary tract*		
Procedural pain	10.3 (3)	6.9 (2)
Urinary tract obstruction	3.4 (1)	3.4 (1)
Urinary tract infection	0 (0)	6.9 (2)
Haematuria	0 (0)	3.4 (1)
Urinary tract pain	0 (0)	3.4 (1)
Catheter Pain	0 (0)	3.4 (1)
*Not related to the urinary tract*	10.3 (3)	6.9 (2)

*SES* safety evaluation set, *fURS* flexible ureteroscopy, *MN* mediNiK^®^

**Table 3:** Procedural difficulty based on a 5-point Likert-scale

FAS-Analysis	fURS + MN (*n* = 23)	fURS (*n* = 17)	*p*-value
Very easy	13% (*n* = 3)	0	0.2942
Easy	13% (*n* = 3)	29.4% (*n* = 5)	
Average	34.8% (*n* = 8)	11.8% (*n* = 2)	
Somewhat difficult	26.1% (*n* = 6)	47.1% (*n* = 8)	
Very difficult	13% (*n* = 3)	11.8% (*n* = 2)	

*n* number of subjects in the treatment group; observations;* (%)* percentage based on subjects of respective treatment group; p-value for total comparison based on Chi-square test, *FAS* full analysis set, *fURS* flexible ureteroscopy, *MN* mediNiK^®^

## Discussion

The increasing prevalence of urolithiasis necessitates more effective and minimally invasive treatment options. While traditional methods such as SWL and PCNL remain widely used, recent advancements in endoscopic techniques have shifted the focus toward more precise and effective procedures, such as fURS, particularly for larger stones [16]. Besides the low invasiveness, a key challenge in treating kidney stones, especially larger ones, is clearing residual stone fragments even under < 1 mm from the pyelon after the procedure, as incomplete removal increases the risk of recurrence up to 50% [8, 9].

The goal of achieving zero residual fragments on postoperative CT scans is essential to prevent the need for reintervention. In this context, a stone-free status defined as Grade A (no detectable residuals on imaging) represents the optimal outcome. Lower grades acknowledge the presence of residual fragments of varying size, which may still be considered acceptable in some settings but do not meet the strict definition of complete stone clearance. As shown by the FLEXOR Group, residual fragments on postoperative imaging often necessitated secondary procedures [17]. The HM explored in this study represents a novel approach aimed at enhancing the extraction of stone fragments during fURS to achieve the highest possible stone-free rate. Comparable concepts, such as fibrin-based gels, have also demonstrated feasibility in fragment retrieval, although they differ in composition and handling [18]. Alternative concepts, such as magnetic hydrogels that enable magnetizable fragment retrieval, have also been described [19]. Together, these developments highlight the interest in innovative adjuncts to improve fragment clearance.

Recent advances reflect two distinct developments: pulse modulation of conventional Ho: YAG systems (e.g., MOSES), which modifies pulse shape to reduce retropulsion and improve energy delivery, and the thulium fiber laser (TFL), a different laser platform characterized by lower peak power and higher repetition rates,, associated with superior dusting [20]. While both can accelerate dusting, they often generate substantial submillimeter debris, leaving a residual fragment burden that underscores the need for adjunctive clearance strategies (e.g., suction or gel-assisted retrieval). However, this progress has paradoxically increased residual fragments, with rates over 25% in some cases [11, 21–23]. Techniques such as the ABC method, which involves injecting the patient’s own blood into the calyx, where the fragments become embedded and can subsequently be removed with a basket, and suction access sheaths have been developed to address this issue, but each has limitations. Additionally suction-assisted techniques have recently been introduced as adjuncts to fURS to improve fragment clearance. It is important to note, however, that these devices differ in their mechanism of action. While the flexible and navigable suction UAS allow to aspirate parallel to the scope fragments smaller than 1 mm [24] direct in scope suction works differently. In those scopes the working channel is used alternatively between irrigation/lasering and suction. Due to the small size of the working channel (3.6–5.1 french), this means that the particle should be around 0.5 mm to allow a good suction [25]. Suction-based techniques, often struggle with larger fragments or irregular shapes and carry a risk of mucosal trauma if not carefully managed [26]. Thus, while new technologies like laser or suction with low-pressure irrigation systems improve visibility and facilitate finer stone dusting, clinical outcomes in terms of SFR often fall short of expectations with SFR remaining below 80% [21, 22, 27, 28]. These findings underscore the need for a more effective method to remove dusted fragments and enhance stone clearance, as current techniques and technologies still fall short in addressing this challenge.

The results of this study suggest that the HM could be a promising alternative. In this first proof-of-concept trial, the application of MN during fURS was safe, with no severe TEAEs observed. Most notably, MN significantly increased the number of retrieved stone fragments, both < 1 mm and > 1 mm, compared to conventional fURS alone. In contrast to the HM, current suction devices are limited in retrieving fragments larger than 1 mm, highlighting a potential advantage of MN. Although operative time was approximately 20 min longer with MN, this difference may decrease with growing experience as the learning curve is overcome. Importantly, the mean duration remained well below the 120-minute safety threshold for fURS, and no increase in complications was observed in this feasibility and safety study. However, given the exploratory design and limited sample size, the trial was not powered for definitive between-group comparisons; thus, differences such as operative time (although statistically significant here) should be interpreted with caution and confirmed in larger, adequately powered prospective studies. Furthermore, the possibility of preventing a second surgery or undermining an early recurrency could benefit a cost reduction on the long term.

Compared to suction-based techniques or the ABC method, the MN method offers distinct advantages. First, it offers a more controlled environment for fragment extraction, reducing the risk of tissue trauma and bleeding while capturing and retaining residual fragments. Moreover, it does not require additional tools or instruments and remains compatible with all flexible ureteroscopes. These qualities make it a viable option for enhancing stone clearance during fURS, potentially reducing the likelihood of recurrence without incurring the complications or additional costs associated with other techniques.

Several limitations should be acknowledged. First, the relatively small sample size restricts the statistical power and generalizability of the findings. Second, as this was a feasibility and safety study, no systematic pre- and postoperative stone assessments were performed, which prevents conclusions about stone-free rates or recurrence. Consequently, the results primarily demonstrate the technical feasibility of fragment extraction rather than long-term clinical efficacy. To address these gaps, larger trials with pre- and postoperative imaging, standardized stone-free rate assessment, and direct comparison with established techniques such as standard fURS are currently underway (ClinicalTrials.gov ID NCT06469736). These studies will be critical for validating our findings and defining the potential role of the hydrogel method in urolithiasis management.

## Conclusion

Overall, HM during fURS proved safe and feasible. As an adjunct to routine fURS, MediNiK^®^ facilitated the retrieval of small fragments and allowed extraction of particles that might otherwise be missed with standard techniques, without increasing the risk of complications. These findings suggest a potential to improve postoperative stone-free rates, but confirmation in larger studies is required to establish whether this translates into reduced recurrence compared with existing approaches.

## Data Availability

No datasets were generated or analysed during the current study.

## Abbreviations

MN: mediNiK^®^
HM: Hydrogel method
SFR: Stone free rate
SoC: Standard of care
fURS: Flexible ureterorenoscopy
SWL: Extracorporeal shock waves
PNL: Percutaneous nephrolithotomy
CIRF: Clinically irrelevant residual fragments
RF: Residual fragments
ABC: Autogenous blood clot technique
SES: Safety evaluation set
FAS: Full analysis set
TEAE: Treatment-emergent adverse events
CT: Computed tomogaphy
UAS: Ureteral access sheath

## References

[1] Stamatelou K, Goldfarb DS (20253) Epidemiology of Kidney Stones. Healthcare (Switzerland) 11. https://pubmed.ncbi.nlm.nih.gov/36766999/, Accessed 16 Aug 2025 10.3390/healthcare11030424 PMC9914194 36766999

[2] Geraghty RM, Jones P, Somani BK (2017) Worldwide Trends of Urinary Stone Disease Treatment over the Last Two Decades: A Systematic Review. J Endourol 31: 547–556. https://pubmed.ncbi.nlm.nih.gov/28095709/, Accessed 16 Aug 2025 10.1089/end.2016.0895 28095709

[3] Abedi G, Monga M (2021) Flexible Ureteroscopy for Treatment of Upper Urinary Tract Calculus. J Endourol 35: S56–S61. https://pubmed.ncbi.nlm.nih.gov/34499545/ Accessed 16 Aug 2025 10.1089/end.2020.1018 34499545

[4] Lildal SK, Andreassen KH, Baard J et al (2020) Consultation on kidney stones, Copenhagen 2019: aspects of intracorporeal lithotripsy in flexible ureterorenoscopy. World J Urol 39. https://pubmed.ncbi.nlm.nih.gov/33067728/ Accessed 16 Aug 2025 10.1007/s00345-020-03481-9 PMC8217045 33067728

[5] Bhojani N, Paonessa JE, El Tayeb MM et al (2018) Sensitivity of Noncontrast Computed Tomography for Small Renal Calculi With Endoscopy as the Gold Standard. Urology 117: 36–40. https://pubmed.ncbi.nlm.nih.gov/29625137/ Accessed 16 Aug 2025 10.1016/j.urology.2018.03.041 29625137

[6] Osman Y, Harraz AM, El-Nahas AR et al (2013) Clinically insignificant residual fragments: An acceptable term in the computed tomography era? Urology 81: 723–726. https://pubmed.ncbi.nlm.nih.gov/23465152/ Accessed 16 Aug 2025 10.1016/j.urology.2013.01.011 23465152

[7] Altunrende F, Tefekli A, Stein RJ et al (2011) Clinically insignificant residual fragments after percutaneous nephrolithotomy: Medium-term follow-up. J Endourol 25: 941–945. https://pubmed.ncbi.nlm.nih.gov/21599528/ Accessed 16 Aug 2025 10.1089/end.2010.0491 21599528

[8] Hein S, Miernik A, Wilhelm K et al (2016) Clinical significance of residual fragments in 2015: impact, detection, and how to avoid them. World J Urol 34: 771–778. https://pubmed.ncbi.nlm.nih.gov/26497824/ Accessed 16 Aug 2025 10.1007/s00345-015-1713-2 26497824

[9] Yeh HT, Wong M (2005) How significant are clinically insignificant residual fragments following lithotripsy? Curr Opin Urol 15: 127–131. https://pubmed.ncbi.nlm.nih.gov/15725937/ Accessed 16 Aug 2025 10.1097/01.mou.0000160628.43860.f9 15725937

[10] Skolarikos AJHNAPA et al (2025) EAU Guidelines. Edn. presented at the EAU Annual Congress Madrid 2025. ISBN 978-94-92671-29-5

[11] Cloutier J, Cordeiro ER, Kamphuis GM et al (2014) The glue-clot technique: a new technique description for small Calyceal stone fragments removal. Urolithiasis 42:441–444 25004802 10.1007/s00240-014-0679-7

[12] Hein S, Schoenthaler M, Wilhelm K et al (2016) Novel Biocompatible Adhesive for Intrarenal Embedding and Endoscopic Removal of Small Residual Fragments after Minimally Invasive Stone Treatment in an Ex Vivo Porcine Kidney Model: Initial Evaluation of a Prototype. Journal of Urolo 196: 1772–1777. https://pubmed.ncbi.nlm.nih.gov/27256206/ Accessed 16 Aug 2025 10.1016/j.juro.2016.05.094 27256206

[13] Hausmann T, Becker B, Gross AJ et al (2021) Novel Biocompatible Adhesive to Remove Stone Dust: Usability Trial in a Kidney Model. J Endourol 35: 1223–1228. https://pubmed.ncbi.nlm.nih.gov/33559523/ Accessed 16 Aug 2025 10.1089/end.2020.0748 33559523

[14] Hein S, Schoeb DS, Grunwald I et al (2018) Viability and biocompatibility of an adhesive system for intrarenal embedding and endoscopic removal of small residual fragments in minimally-invasive stone treatment in an in vivo pig model. World J Urol 36: 673–680. https://pubmed.ncbi.nlm.nih.gov/29368229/ Accessed 16 Aug 2025 10.1007/s00345-018-2188-8 29368229

[15] Amiel T, Straub M, Grunwald I (2022) First in-human application of a novel hydrogel for the removal of residual kidney stone fragments. Eur Urol Open Sci 39:S145–S146

[16] Ates T, Sukur IH, Ok F et al (2022) Global research trends in minimally invasive treatments for kidney stones: A bibliometric analysis (2015–2024). Urolithiasis 53. https://pubmed.ncbi.nlm.nih.gov/40522467/ Accessed 16 Aug 2025 10.1007/s00240-025-01785-2 40522467

[17] Gauhar V, Chew BH, Traxer O et al (2023) Indications, preferences, global practice patterns and outcomes in retrograde intrarenal surgery (RIRS) for renal stones in adults: results from a multicenter database of 6669 patients of the global FLEXible ureteroscopy Outcomes Registry (FLEXOR). World J Urol 41: 567–574. https://pubmed.ncbi.nlm.nih.gov/36536170/ Accessed 16 Aug 2025 10.1007/s00345-022-04257-z 36536170

[18] Yu Y, Xi H, Chen Y et al (2022) Fibrin gel-assisted stone extraction in retrograde intrarenal surgery. BJU Int 129:170–173 34856054 10.1111/bju.15651 PMC9300148

[19] Ge TJ, Roquero DM, Holton GH et al (2023) A magnetic hydrogel for the efficient retrieval of kidney stone fragments during ureteroscopy. Nat Commun 14:3711 37349287 10.1038/s41467-023-38936-1 PMC10287666

[20] Chandramohan V, Swamy PMS, Ramakrishna P et al (2023) Ureteroscopic lithotripsy by thulium fiber laser versus holmium laser: A single-center prospective randomized study. Urol Ann 15: 285–288. https://pubmed.ncbi.nlm.nih.gov/37664089/Accessed 16 Aug 2025 10.4103/ua.ua_115_22 PMC10471822 37664089

[21] Chai CA, Inoue T, Somani BK et al (2024) Comparing thulium fiber versus high power holmium laser in bilateral same sitting retrograde intrarenal surgery for kidney stones: results from a multicenter study. Investig Clin Urol 65:451 39249917 10.4111/icu.20240185 PMC11390269

[22] Ulvik Ø, Æsøy MS, Juliebø-Jones P et al (2022) Thulium fibre laser versus holmium:yag for ureteroscopic lithotripsy: outcomes from a prospective randomised clinical trial. Eur Urol 82:73–79 35300888 10.1016/j.eururo.2022.02.027

[23] Castellani D, Fong KY, Lim EJ et al (2023) Comparison between holmium:yag laser with MOSES technology vs thulium fiber laser lithotripsy in retrograde intrarenal surgery for kidney stones in adults: A propensity Score–matched analysis from the flexible ureteroscopy outcomes registry. J Urol 210:323–330 37126223 10.1097/JU.0000000000003504

[24] Gauhar V, Ong CS-H, Traxer O et al (2023) Step-by-step guide to flexible and navigable Suction ureteric access sheath (FANS). Urol Video J 20:100250

[25] Okhunov Z, Baldwin EA, Jhang D et al (2025) Which sucks more? Comparison of two novel direct-in-scope Suction ureteroscopes to a conventional ureteroscope. World J Urol 43:495 40820048 10.1007/s00345-025-05768-1 PMC12358325

[26] Gauhar V, Traxer O, Castellani D et al (2023) A feasibility study on clinical Utility, efficacy and limitations of 2 types of flexible and navigable Suction ureteral access sheaths in retrograde intrarenal surgery for renal stones. Urology 178:173–179 37328010 10.1016/j.urology.2023.05.032

[27] Kwok J-L, Somani B, Sarica K et al (2024) Multicenter outcome analysis of different sheath sizes for flexible and navigable Suction ureteral access sheath (FANS) ureteroscopy: an EAU endourology collaboration with the global FANS study group. Urolithiasis 52:162 39545972 10.1007/s00240-024-01662-4

[28] Giulioni C, Castellani D, Falsetti F et al (2025) Single-use versus reusable flexible ureterorenoscopes with FANS: a multicenter propensity-matched analysis of outcomes in a large series from the EAU-Endourology section and FANS collaborative group. World J Urol 43:399 40569462 10.1007/s00345-025-05769-0

